# Supplementary material for: Direct observation of photoinduced sequential spin transition in a halogen-bonded hybrid system by complementary ultrafast optical and electron probes
Source: Nat Commun. 2024 Jun 4;15:4604. doi: 10.1038/s41467-024-48529-1 (PMC11150260; doi:10.1038/s41467-024-48529-1)
Supplement: Supplementary file 1 — Supplementary Information [file 41467_2024_48529_MOESM1_ESM.pdf]

Supplementary Information

Direct observation of photoinduced sequential spin transition in a halogen-bonded hybrid system by complementary ultrafast optical and electron probes

Jiang *et al.*

Contents

Supplementary Method 1 | Temperature dependence measurement. 2
Supplementary Method 2 | Polarization dependence of the electron diffraction experiments. 4
Supplementary Method 3 | Fluence dependence measurement of transient absorption measurement. 6
Supplementary Method 4 | Relaxation process analysis. 9
Supplementary Method 5 | Debye Waller effect correction of ultrafast electron diffraction data and temperature estimation 11
Supplementary Method 6 | Time-dependent model of atomic motions. 14
Supplementary Method 7 | The interaction energy calculation 17
Supplementary Discussion 1 | Photoinduced dynamics of Fe(III) spin crossover 18
Supplementary Discussion 2 | Assignment of spectral features of mid-infrared vibrational spectroscopy at ~1350 and 1450 cm^{-1}. 18
Supplementary Discussion 3 | Impact of volume expansion in dynamics 20
Supplementary Discussion 4 | Influence of volume expansion in UED data 21
Supplementary References 23

Supplementary Method 1 | Temperature dependence measurement.

The hybrid complex $[Fe(Iqsal)_2][Ni(dmit)_2]\cdot CH_3CN\cdot H_2O$ (**1**) (Iqsal = 5-iodo-*N*-(8'-quinolyl)-salicylaldiminate, dmit = 1,3-dithiole-2-thione-4,5-dithiolate) was reported to have thermally-induced phase transition between the low-temperature (LT) and high-temperature (HT) phases[1]. The thermally-induced phase transition of **1** has been studied by the magnetic susceptibilities and Mössbauer spectroscopy. These measurements show the phase transition was abrupt with transition temperature (T_c = 150 K)[1]. In this work, the temperature dependence measurement of optical absorption spectra and electron diffraction of **1** was performed before the time-resolved measurements to ensure the sample is in the LT phase.

In Fig. 2a, the optical density (OD) was measured at different temperatures (77 and 290 K) using the same setups used for time-resolved transition absorption spectroscopy (TA). We used conduction type cryostat, Microstat (Oxford instruments). There is a clear change of OD (ΔOD) between the LT and HT phases. Supplementary Fig. 1d and 1e show the temperature dependence of ΔOD and ΔOD at 480 nm with polarization parallel to the b-axis ($E \parallel$ b), respectively. We have determined that T_c of the thin sample is approximately 140 K from Supplementary Fig 1e, which is close to the reported T_c of 150 K of the bulk single crystal[1].

In the electron diffraction (ED) experiment, static diffraction patterns were collected at different temperatures, from 120 to 230 K with the same setup as the ultrafast electron diffraction (UED) measurement. We performed simulations to show the differences in diffraction patterns between the LT and HT structures, as determined through X-ray diffraction (Supplementary Fig. 1a). These simulated changes in Bragg peak intensities due to thermally-induced phase transition align closely with the experimental changes (Supplementary Fig. 1b). In Supplementary Fig. 1c, the relative changes of selected Bragg peaks intensity at different temperatures are shown. The T_c appears to be around 180 K, which is a little higher than reported in the literature[1] and our optical measurement shown in Supplementary Fig. 1. We suspect this discrepancy to be due to a calibration error due to poor thermal contact between the temperature sensor in the ED setup and the sample holder so that the temperature readout does not reflect the actual temperature of the sample. Nevertheless, it is safe to say the temperature for the UED experiments (125 K) was below T_c and the sample was in the LT phase.

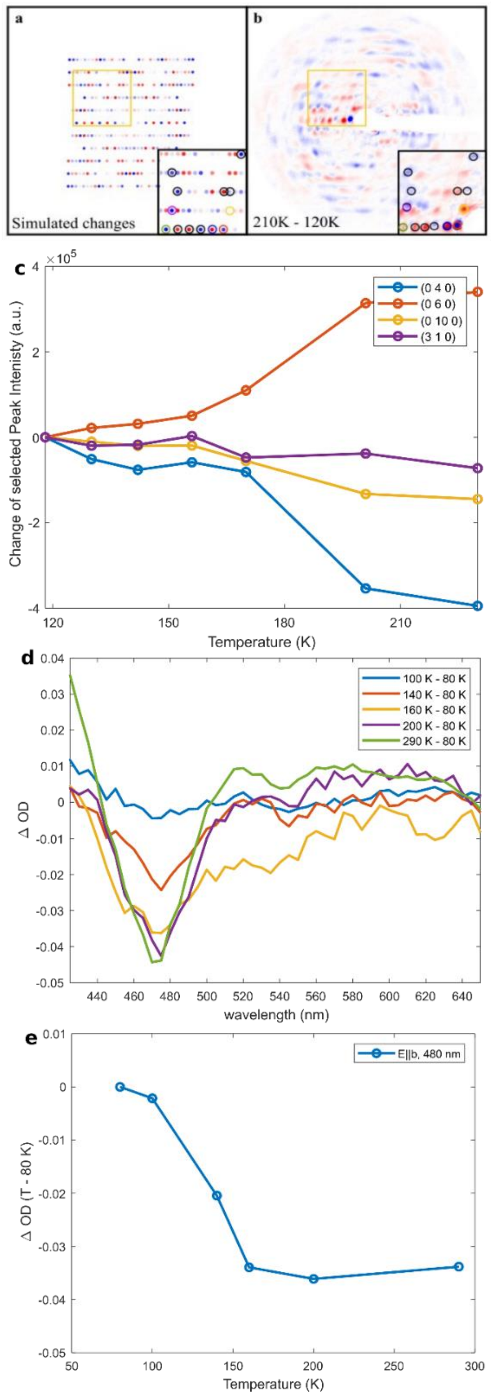

Supplementary Fig. 1 | Temperature dependence studies on ultrathin samples with UED and TA. a) Simulated differences in diffraction patterns between the low-temperature and high-temperature structures from literature[1]. The inset shows the selected area of the diffraction pattern. b) Experimental thermally-induced changes in our UED setup c) Relative changes of selected Bragg peaks. d) Temperature dependence of the ΔOD spectra relative to the OD at 80 K. e) Line trace of (d) at 480 nm.

Supplementary Method 2 | Polarization dependence of the electron diffraction experiments. Supplementary Fig. 2a shows the OD spectra for $E \parallel b$ and $E \parallel c$ polarization on the *bc* plane of the thin sample. The spectra show a strong absorption at 400 nm with both $E \parallel b$ and $E \parallel c$ polarization at 290 and 100 K.

Supplementary Fig. 2b and 2c show the oscillator strength of the electronic transition in the high-spin (HS) state of the isolated $[Fe(Iqsal)_2]^+$ cation and the $[Ni(dmit)_2]^-$ anion. The directions of cation and anion are the same as in the crystal. Summation of the oscillator strength of the isolated ions show the good correspondence to the measured OD spectra, especially the anisotropy. The $[Fe(Iqsal)_2]^+$ cation shows a strong absorption at 400 nm with both $E \parallel b$ and $E \parallel c$ polarization, and this absorption is assigned to the ligand-to-metal charge transfer (LMCT) transition. On the other hand, the calculated results of the isolated $[Ni(dmit)_2]^-$ anion has little absorption at 400 nm with $E \parallel c$. Therefore, it is possible to excite only the $[Fe(Iqsal)_2]^+$ cation in the **1** sample at 400 nm with $E \parallel c$ polarization and observe how the $[Ni(dmit)_2]^-$ anion response to the photoinduced SCO in the $[Fe(Iqsal)_2]^+$ cations.

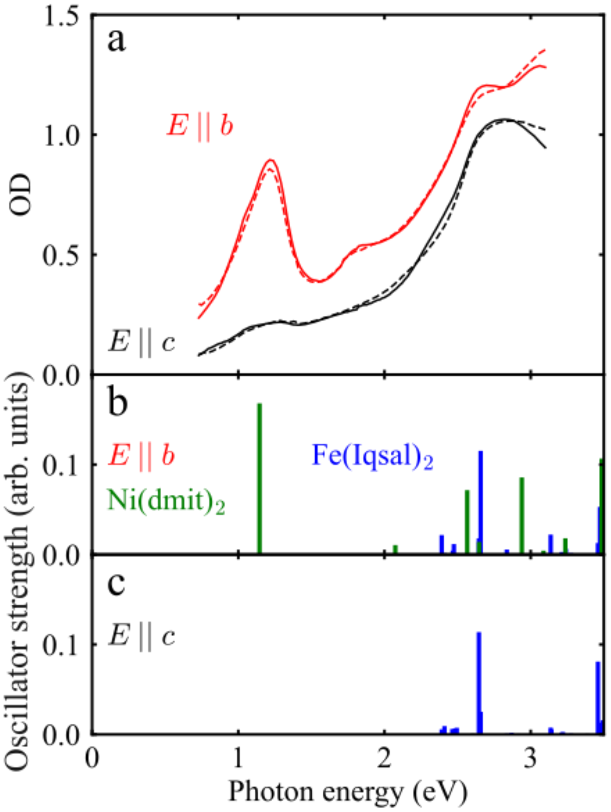

Supplementary Fig. 2 | Optical spectrum of 1 and oscillator strength of the $[Fe(Iqsal)_2]^+$ cation and the $[Ni(dmit)_2]^-$ anion. a, Optical density of the **1** thin sample at 290 K (dashed lines) and 100 K (solid lines) with $E||b$ (red) and $E||c$ (black). The **1** sample has strong absorption at 400 nm with both $E||b$ and $E||c$. **b,** Oscillator strength of the $[Fe(Iqsal)_2]^+$ cation and the $[Ni(dmit)_2]^-$ anion in the 1 sample with $E||b$. **c,** Oscillator strength of the $[Fe(Iqsal)_2]^+$ cation and the $[Ni(dmit)_2]^-$ anion in the 1 sample with $E||c$. By comparing the calculations and the optical density

measurement, it is suggested that with *E*||*b* polarization, both the $[Fe(Iqsal)_2]^+$ cation and $[Ni(dmit)_2]^-$ anion are excited by the pump laser. With *E*||*c* polarization, only the $[Fe(Iqsal)_2]^+$ cation is excited by the pump laser.

To confirm our excitation conditions and the validity of our results, we first performed polarization dependence studies using UED to measure the mean of absolute relative intensity changes (MARIC) of most diffraction peaks $\frac{1}{N}\sum_{i=1}^{N}\left|\frac{I_i - I_{i_0}}{I_{i_0}}\right|$ (where N is the total number of diffraction peaks, I_i is the intensity of i-th diffraction peaks after photoexcitation, I_{i_0} is the intensity for the i-th peak without photoexcitation) at +5 ps with different polarization of pump laser. At this time delay, the difference signals are stronger for a clear comparation. Supplementary Fig. 3 shows the MARIC of most Bragg peaks' intensities reach a maximum at 0-degree (*E* || *b*) and a minimum at 90-degree (*E* || *c*) by changing angle of polarization of the pump laser with the half wave plate. The orientation of the crystal was determined by the diffraction pattern. The UED experiment results suggest the sample **1** has a strong absorption with *E* || *b* and a weak absorption with *E* || *c*. These polarization measurements using UED are consistent with the theory calculations and OD measurements as shown in Supplementary Fig. 2.

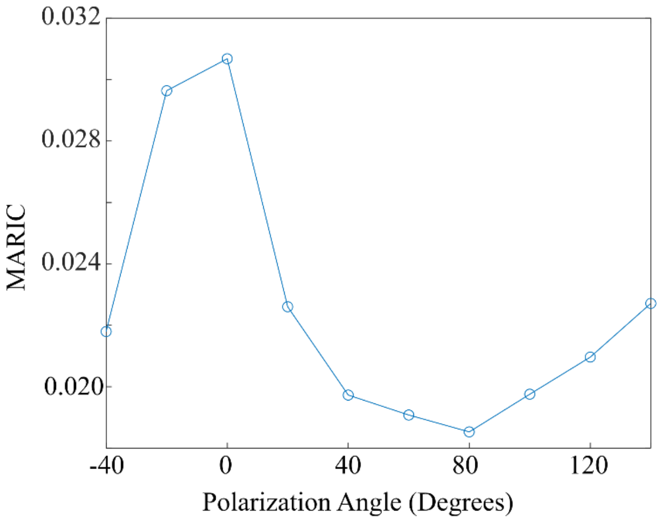

Supplementary Fig. 3 | Mean Absolute Relative Intensity Change (MARIC) of most Bragg peaks at +5 ps with different polarization of pump laser. *E*||*b* is at 0° and *E*||*c* is at 90°.

Supplementary Fig. 4a and 4b show difference maps of Bragg peak intensities at time delay of +5 ps with *E* || *b* and *E* || *c* polarization of the pump laser, respectively. It is important to notice that changes of intensities with *E* || *b* polarization are very similar to the changes of intensities due to thermally-induced phase transition (Supplementary Fig. 4c). This suggests that after pumping with *E* || *b* polarization, **1** reaches a HT-like state [HS state of the $[Fe(Iqsal)_2]^+$ cation with weak dimerization (WD) of the $[Ni(dmit)_2]^-$ anion] in ultrafast +5 ps timescale.

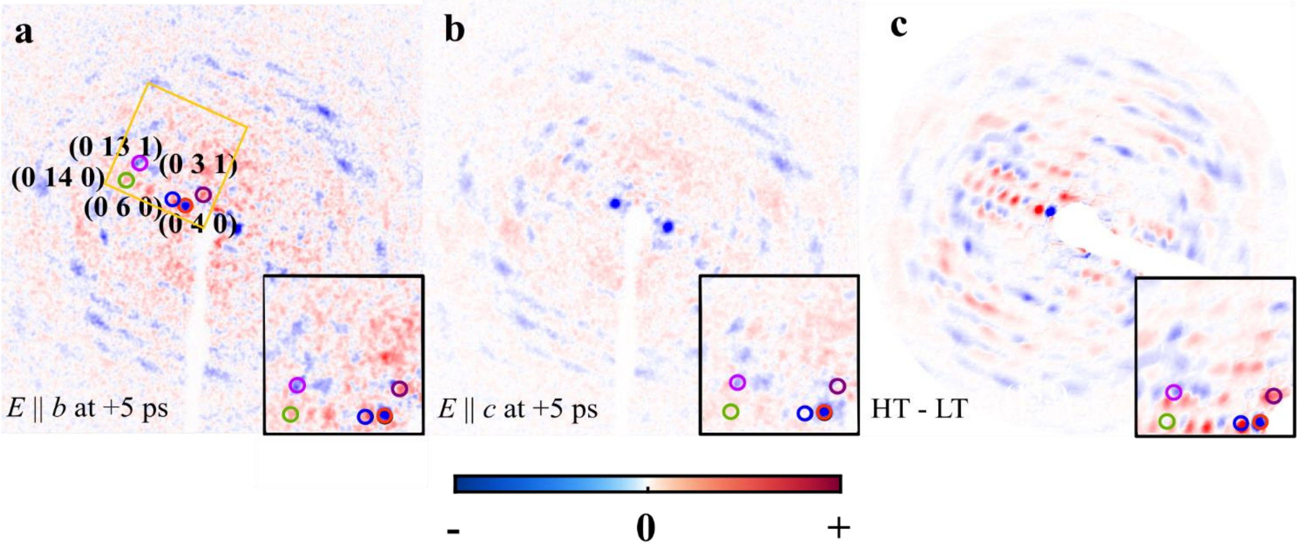

Supplementary Fig. 4 | Changes in Bragg peak intensities with excitation of *E*||*b* and *E*||*c* polarization in ultrafast timescale. a, The changes of intensities with *E*||*b* at +5 ps. **b**, The changes of intensities with *E*||*c* at +5 ps. **c,** Thermally-induced changes. The insets show magnified images of the diffraction patterns in a selected area indicated by yellow rectangle in panel **a** for a clear comparison.

The change of Bragg peak intensities after pumping with *E* || *c* polarization shows different patterns between at +5 ps (Supplementary Fig. 4b) and thermally-induced changes (Supplementary Fig. 4c). In insets of Supplementary Fig. 4b and 4c, the selected peaks have different changes in intensities with *E* || *c* excitation and thermally-induced phase transition. In particular, the peak (0 3 1) has a positive change of the diffraction intensity with thermally-induced phase transition but has a weak negative change with *E* || *c* excitation as it was shown in Figure 4e. On the other hand, peak (0 4 0) still shows similar negative changes. These differences suggest a distant structure of the excited state at +5 ps with *E* || *c* and of the HT phase. In Fig. 4g, we used a structural refinement algorithm to show that this intermediate state with *E* || *c* at +5 ps is at HS state with strong dimerization (SD) state.

Considering the above discussions about excitation polarization dependence of the diffraction pattern at +5 ps, it is a natural assignment that only the $[Fe(Iqsal)_2]^+$ cation is excited by the pump laser with *E* || *c* polarization and both the $[Fe(Iqsal)_2]^+$ cation and $[Ni(dmit)_2]^-$ anion are excited by the pump laser with *E* || *b*. Our polarization dependent measurement shows that the pump laser with different polarization can trigger different photoinduced dynamics of **1**.

Supplementary Method 3 | Fluence dependence measurement of transient absorption measurement.

In the TA measurement, the fluence dependence was measured 2 ps after photoexcitation with fluencies ranging from 0.1 to 6.11 mJ/cm^2. In this work, we used SVD to filter the data which helps to remove noise to show a clear correlation between ΔOD and the fluence. Supplementary Fig. 5a shows SVD filtered ΔOD spectra at a range of fluences, Supplementary Fig. 5b shows

residuals of the SVD filtering (raw ΔOD minus SVD filtered ΔOD), which shows negligible difference and assures the validity of SVD filtered ΔOD. In Supplementary Fig. 5c, we present the fluence dependence of SVD filtered ΔOD at 450 nm which is linear until 3.67 mJ/cm^2.

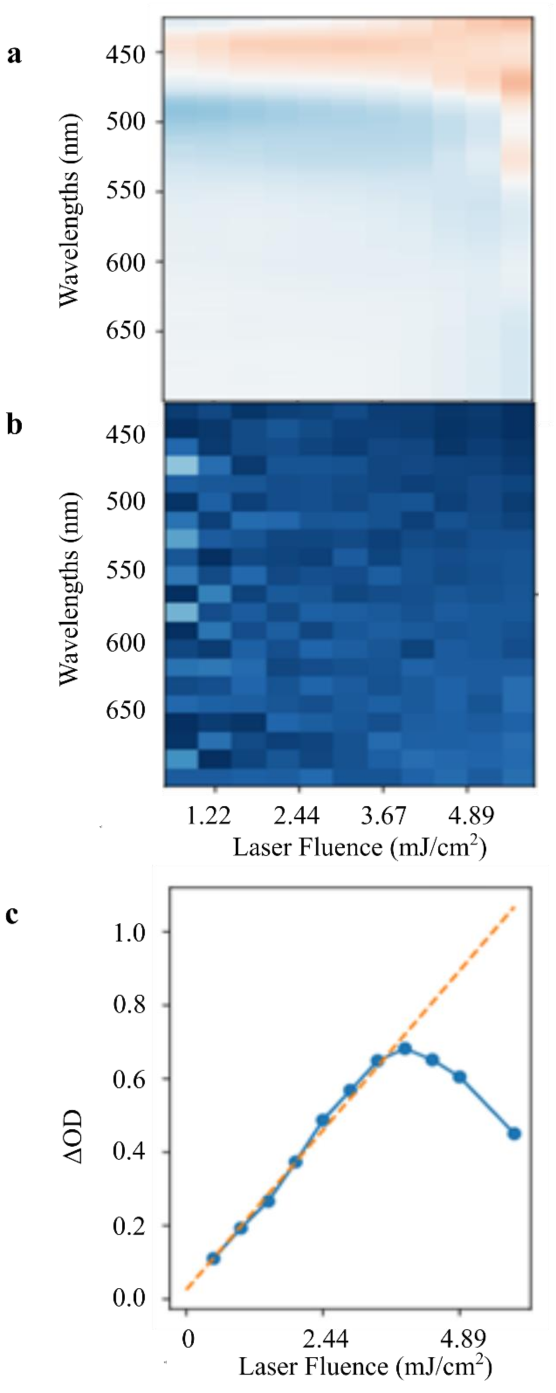

Supplementary Fig. 5 | Fluence dependence measurements of transient absorption measurement. **a**, Transient absorption (ΔOD) spectra recorded with varying laser fluence at +2 ps after photoexcitation, filtered by SVD. **b**, Residuals of the SVD filtered ΔOD. **c**, ΔOD at 450 nm at +2 ps as a function laser fluence.

With the fluence used in the experiment, as shown in Supplementary Fig. 6, it enables hours of data collection without sample damage.

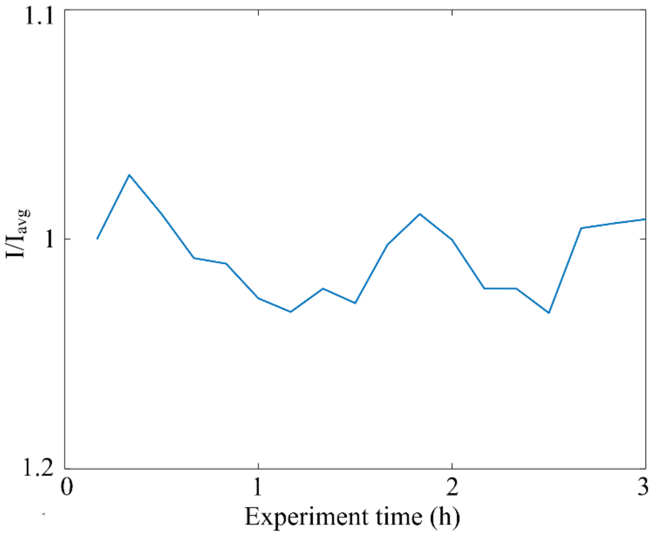

Supplementary Fig. 6 | Relative change of the overall diffraction patterns intensity during a 3-hour collection of the UED data to the overall diffraction patterns intensity in the beginning of the measurement. 400 nm laser with 0.51 mJ/cm^2 fluence was used to excite the $[Fe(Iqsal)_2]^+$ cations. This fluence is below the sample damage threshold and enables hours of pump-probe with 125 Hz without change of all Bragg peak intensity, reflecting no sample damage during this 3-hour data collection.

Supplementary Method 4 | Relaxation process analysis.

In this work, we used a global fitting procedure with using Supplementary Equation 1 to analyze the time delay (t) dependence in TA, MIR, and UED dataset with sharing the relaxation time τ_j.

$$f(t)=\sum_{j=1}^{Q}\left[a_j\left(H(t)e^{-t/\tau_j}\right)*IRF(t)\right] \qquad \text{Supplementary Equation (1)}$$

, where Q, a_j, $H(t)$, $IRF(t)$, and τ_j are number of relaxation processes, amplitudes, a Heaviside step function, and an instrument response function (a Gaussian function centered at $t = 0$), respectively.

Before performing the global fitting of UED and TA datasets, we used a singular value decomposition (SVD) to extract components of t-dependent data and remove noise[2,3]. After adopting SVD, we could reduce the number of t-dependent curves to four relaxation processes. Hereafter, we call the summation of reduced set of temporal profiles "SVD filtered data".

We performed global fitting for the reduced set of temporal profiles. Especially for the SVD filtered TA data [$\Delta A(t,\lambda)$, λ: wavelength (nm)], we made decay associated spectra [DAS: $D_i(\lambda)$] as defined in Supplementary Equation 2[4,5].

$$\Delta A(t,\lambda)=\sum_{j=1}^{Q}D_j(\lambda)\left[\left(H(t)e^{-t/\tau_j}\right)*IRF(t)\right] \qquad \text{Supplementary Equation (2)}$$

For time traces obtained in MIR experiment, we performed global fitting of the raw data of relative reflectivity change without performing SVD.

In Supplementary Fig. 7, we show the DAS of $[Fe(Iqsal)_2][Ni(dmit)_2]\cdot CH_3CN\cdot H_2O$ (**1**) deduced from the fitting analysis from the short-time time-resolved transition absorption spectroscopy (TA) data in Fig. 2c. In Supplementary Fig. 8, we present the DAS of **1** deduced from fitting analysis of time-resolved TA data in Fig. 2d.

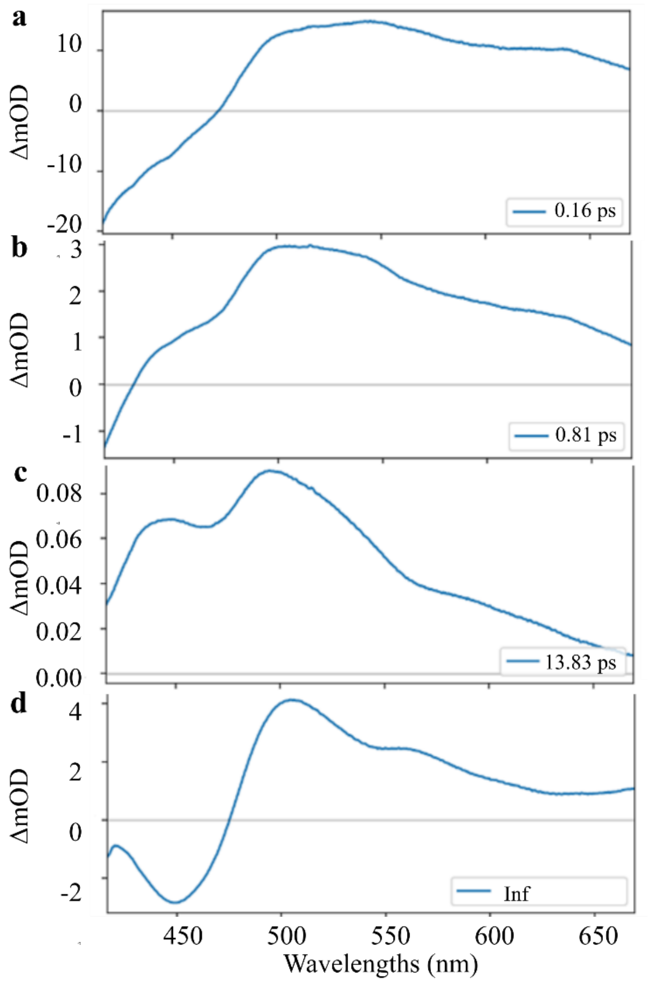

Supplementary Fig. 7 | Decay-associated spectra (DAS) of [Fe(Iqsal)$_2$][Ni(dmit)$_2$]·CH_3CN·H_2O (1) deduced from the fitting analysis from the short-time time-resolved transition absorption spectroscopy (TA) data in Fig. 2c. a, The first component with 0.16 ps time constant. It is assigned to be ligand-to-metal-charge-transfer (LMCT) of Fe(III) spin crossover. **b,** The second component with 0.81 ps time constant is assigned to be intramolecular vibrational energy redistribution (IVR) dynamics of Fe(III) spin crossover. **c,** The third component with13.83 ps time constant. **d,** The last component with infinite on this time scale. This is most likely the result of transient lattice heating.

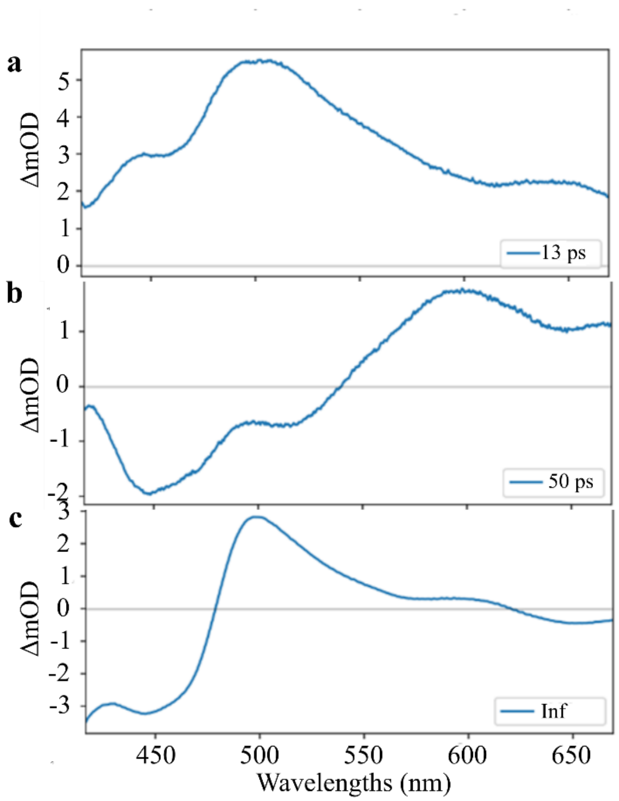

Supplementary Fig. 8 | DAS of 1 deduced from fitting analysis of time-resolved TA data in Fig. 2d. **a,** The first component with 13 ps time constant. **b,** The second component with 50 ps time constant. **c,** The last component with infinite on this time scale. This is most likely the result of the high spin (HS)⟶ low spin (LS) relaxation and transient lattice heating.

Supplementary Method 5 | Debye Waller effect correction of ultrafast electron diffraction data and temperature estimation

In the photoinduced changes of Bragg intensities measured at +5 (after initial SCO) and +100 ps (after dimer softening) time delay, we notice that many high order Bragg spots exhibit relatively stronger decrease in intensity than the thermal induced changes in Fig. 4c and 4d. This indicates a Debye Waller (DW) effect in the timescale of our measurements. The DW effect is a decrease in diffraction intensity caused by thermal motion, related to the random mean square displacement of atoms from their average position. In this UED experiment, it is induced by the pump laser, and it has been widely observed in many UED studies.

The DW effect provides a snapshot of the temperature evolution of the sample by fitting the changes of Bragg peaks at a given time point to a model based on the DW effect. This calculation is based on the photo-Wilson plot (natural logarithm of the quasi-steady-state intensity of 100 Bragg peaks vs d-spacing squared) (Supplementary Equation 3)[6,7].

$$\ln[\,I(t)/I_0\,] = -4\pi^2 \frac{1}{d^2} \langle \delta u^2(t) \rangle / 3 \qquad \text{Supplementary Equation (2)}$$

where I(t) is the intensity of the diffraction peak at a given time t after photoexcitation. I_0 is the intensity before excitation, d is the distance between crystal planes, and $\langle \delta u^2(t) \rangle$ is the mean square atomic displacement.

To calibrate the change in temperature due to the DW effect, we extracted the isotropic/equivalent atomic displacement parameters ($U_{\mathrm{iso/eq}} = <u^2>/3$) from the crystal information files reported at four distinct temperatures[1] and fitted the mean value, <U>, as a function of temperature, yielding a value of 4.3 x 10^{-4} Å^2/K, in Supplementary Fig. 9a. The uncertainty in this value is large, around 60%, but at least provides an independent estimate of the temperature change. Subsequently, Supplementary Fig. 9b and 9c shows the fitting outcomes of the photo-Wilson plot at two specific time points. The fitted slope reflects the $\langle \delta u^2(t) \rangle$ derived from experimental data. Then we fitted the photo-Wilson plots over the whole-time range and deduced the temperature evolution of the sample (Supplementary Fig. 9d and Supplementary Fig. 9e). Following the initial photoexcitation, the overall sample experiences a temperature jump of 6 ± 4 K, reaching a maximum temperature jump of 17 ± 10 K after the dimer softening process.

After the calculation of the DW temperature, the DW contributions to low-order Bragg peaks are found to be less than 0.1%, which is much smaller than the changes at the thermal phase transition. We calculated contributions of DW effect on every Bragg peak intensity and eliminated the contribution of DW effect from the obtained data to only show the changes of the photoinduced sequential spin transition in **1**. In the later modelling and other calculations, the DW effect has been removed.

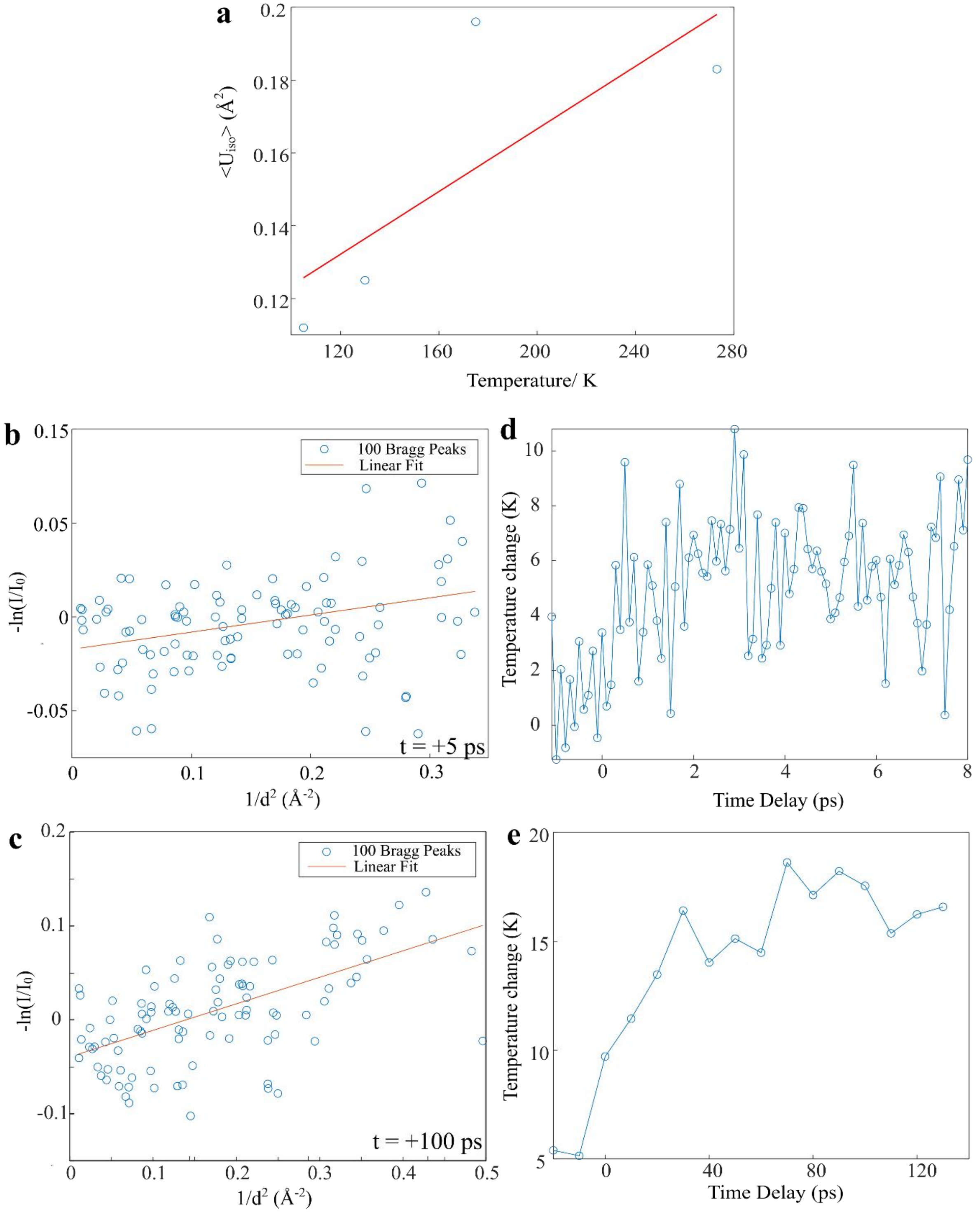

Supplementary Fig. 9 | Debye Waller effect calculation for UED data. a) Isotropic atomic displacement parameter $\langle U_{iso} \rangle$ vs four distinct temperature. Red line is linear fit. b) Natural logarithm of the quasi-steady-state intensity vs 1/d-spacing squared and fitted the result of the calculation at +5 ps (after initial SCO). Red line is linear fit. c) Natural logarithm of the quasi-

steady-state intensity vs 1/d-spacing squared and fitted the result of the calculation at +100 ps (after the reserves dimerization). Red line is linear fit. d) Temperature evolution of the sample from −1 to +8 ps. e) Temperature evolution of the sample from −10 to +130 ps.

Supplementary Method 6 | Time-dependent model of atomic motions.
Both the LT and HT phases are found in the $P2_1/c$ space group[1], with none of the atoms lying on symmetry elements, therefore the N atoms in the unit cell are described by $N/4$ atoms in the asymmetric unit. This is composed of one $[Fe(Iqsal)_2]^+$ cation, one $[Ni(dmit)_2]^-$ anion, one CH_3CN and one H_2O molecule. The main noteworthy feature of the symmetry is that the dimers of $[Ni(dmit)_2]^-$ anions are related by an inversion operation at the midpoint of the interatomic Ni-Ni vector. Since the symmetry remains unchanged during the thermal phase transition, we assume the same symmetry for the ultrafast dynamics.

From the UED data, we chose 80 Bragg peak changes and 112 time points from time delay scans with different step sizes. A SVD filter with the first ten components is used for these Bragg peak changes before the parameterized model fitting. We used a parameterized molecular model and a similar procedure to that used previously to interpret UED data in some other molecular crystals, including two Fe(II) spin crossover systems[8–10]. The workflow is summarized in Supplementary Fig. 10. This method involves identifying possible key modes, generating a set of structures by displacement along these modes, calculating their associated structure factors and intensity changes and calculating the correlation between these and the experimentally observed data for each time delay. The correlation between the simulated and experimental data was calculated using the Pearson correlation coefficient as shown:

$$\mathrm{PC}_{\mathrm{sim,exp}}(\mathbf{p},t)=\frac{\mathrm{cov}(\eta_{\mathrm{sim}}(\mathbf{p},\mathbf{q}_\mathrm{j}),\eta_{\mathrm{exp}}(t,\mathbf{q}_\mathrm{j}))}{\sigma_{\mathrm{sim}}\sigma_{\mathrm{exp}}} \qquad \text{Supplementary Equation (3)}$$

$$\eta_{\mathrm{sim}}\left(\mathbf{p},\mathbf{q}_\mathrm{j}\right)=\frac{\mathrm{F}_{\mathrm{sim}}\left(\mathbf{p},\mathbf{q}_\mathrm{j}\right)^2}{\left(\mathrm{F}_{\mathrm{off}}\left(\mathbf{q}_\mathrm{j}\right)\right)^2}-1 \qquad \text{Supplementary Equation (4)}$$

$$\eta_{\mathrm{exp}}\left(t,\mathbf{q}_\mathrm{j}\right)=\frac{\mathrm{I}_{\mathrm{exp}}\left(t,\mathbf{q}_\mathrm{j}\right)}{\mathrm{I}_{\mathrm{off}}(\mathbf{q}_\mathrm{j})}-1 \qquad \text{Supplementary Equation (5)}$$

$$\mathrm{cov}(\eta_{\mathrm{sim}}\left(\mathbf{p},\mathbf{q}_\mathrm{j}\right),\eta_{\mathrm{exp}}\left(\mathrm{t},\mathbf{q}_\mathrm{j}\right))=\frac{1}{\mathrm{M}}\sum_{\mathrm{j}=1}^{\mathrm{M}}\left(\eta_{\mathrm{sim}}\left(\mathbf{q}_\mathrm{j}\right)-\bar{\eta}_{\mathrm{sim}}\left(\mathbf{q}_\mathrm{j}\right)\right)\left(\eta_{\mathrm{exp}}\left(\mathbf{q}_\mathrm{j}\right)-\bar{\eta}_{\mathrm{exp}}\left(\mathbf{q}_\mathrm{j}\right)\right)$$

Supplementary Equation (7)

$$\sigma_{\mathrm{sim}}=\sqrt{\frac{1}{M}\sum_{j=1}^{M}\left(\eta_{\mathrm{sim}}\left(\mathbf{q}_j\right)-\bar{\eta}_{\mathrm{sim}}\left(\mathbf{q}_j\right)\right)^2} \quad \text{Supplementary Equation (6)}$$

$$\sigma_{\mathrm{exp}}=\sqrt{\frac{1}{M}\sum_{j=1}^{M}\left(\eta_{\mathrm{exp}}\left(\mathbf{q}_j\right)-\bar{\eta}_{\mathrm{exp}}\left(\mathbf{q}_j\right)\right)^2} \quad \text{Supplementary Equation (7)}$$

PC is Pearson correlation coefficient; **p** is a vector of parameter values to atomic coordinates; η is response ratio (relative intensity change for a given reflection); t is time delay point; **q** is reciprocal lattice vector; j are the indices of the sampled Bragg peaks; F is the structure factor; cov is the covariance; M is the total number of Bragg peaks used in the analysis.

During the modelling, we adopted the following basic assumptions:

1. Fe(III) centers in the $[Fe(Iqsal)_2]^+$ cation were fixed at their position found in the LT crystal structure.
2. The unit cell parameters were fixed to those in the LT structure.

In order to parameterize the motion, the Cartesian coordinates of the HT structure ($\mathbf{x}^{HT}$) were converted into the LT unit cell to give a modified HT structure (**x**'): The coordinates of atoms in the $[Fe(Iqsal)_2]^+$ cation were shifted by the difference between the Fe atom positions at LT and HT as below, where $\boldsymbol{x}_{Fe}$ are the Cartesian coordinates of the Fe atom in either the HT or LT unit cell, as specified by the superscript:

$$\mathbf{x}'_{\mathrm{Fe(Iqsal)_2}}=\mathbf{x}^{\mathrm{HT}}_{\mathrm{Fe(Iqsal)_2}}+\mathbf{x}^{\mathrm{LT}}_{\mathrm{Fe}}-\mathbf{x}^{\mathrm{HT}}_{\mathrm{Fe}} \quad \text{Supplementary Equation (8)}$$

Similarly, the atom positions for the dimers of $[Ni(dmit)_2]^-$ anions in the HT structure were shifted so that their mid-point coincided with the inversion symmetry element in the LT unit cell. With one exception (the dimer expansion), the parameterization was performed by defining a set of atoms as belonging to a group and linearly interpolating their positions between the LT and modified HT structures:

$$\mathbf{x}^{\mathrm{ES}}_{\mathrm{g}}=\mathbf{p}_{\mathrm{g}}\mathbf{x}'_{\mathrm{g}}+(1-\mathbf{p}_{\mathrm{g}})\mathbf{x}^{\mathrm{LT}}_{\mathrm{g}} \quad \text{Supplementary Equation (9)}$$

where g is the index of the group and $\mathbf{p}_g$ is the parameter determining the similarity of the excited state (ES) to the modified high temperature structure and $\mathbf{x}_g$ are the coordinates of the atoms in group g.

The choice of groups was guided by previously reported works on the structural dynamics of SCO systems, the crystal symmetry to describe the dimerization and our knowledge of the LT to HT phase transition. In this regard, the following facts were critical:

1. The system is a multifunctional hybrid system containing the $[Fe(Iqsal)_2]^+$ cation and the $[Ni(dmit)_2]^-$ anion, which are the critical components of the system.

2. The Fe–ligand expansion is the direct structural result of the SCO on the $[Fe(Iqsal)_2]^+$ cation due to a weaker Fe–N and Fe-O bonding in the HS state. Previous studies on SCO materials have shown that the atomic motion during SCO was highly correlated with the change in electron distribution of the metal center[6,8].

3. During the thermally-induced phase transition, the paired $[Ni(dmit)_2]^-$ anions rearrange by slipping along the molecular long axis with the $[Ni(dmit)_2]^-$ anions being related by an inversion operation[1].

The modelling therefore revolved around two independent groups, the $[Fe(Iqsal)_2]^+$ cation and the $[Ni(dmit)_2]^-$ anion and followed the procedure shown in Extended Figure 14.

As well as defining these groups, we tested adding additional parameters to the model as outlined below:

1. For the $[Fe(Iqsal)_2]^+$ cation, we tested breaking it into sub groups.

    a. We divided the $[Fe(Iqsal)_2]^+$ cation into two structural groups, the coordination octahedron and ligands. The change in these groups is involved in the elongation of coordination bonds and the structural rearrangement of the ligands, respectively.

    b. We also defined the iodine atoms on the ligand as an independent group from the rest of the $[Fe(Iqsal)_2]^+$ cation (all atoms on the $[Fe(Iqsal)_2]^+$ cation).

    In both cases, the resulting parameters were highly correlated, indicating that the motion of these groups was also correlated so these parameters were combined into a single group.

2. We examined changes in the distances between the two $[Ni(dmit)_2]^-$ anions. Employing a linear displacement of the $[Ni(dmit)_2]^-$ anion along the midpoint of the Ni-Ni inter-atomic vector, we altered the interplane distance between the two molecular planes. During the refinement, this linear displacement remained at zero, indicating the absence of significant changes in the displacement of the two $[Ni(dmit)_2]^-$ anions. This outcome aligns with the observation that in the thermally-induced phase transition the spacing between the dimers remains approximately constant and is not the cause of the loss of spin paring.

3. The co-crystallised CH_3CN and H_2O solvent molecules were defined as an additional structural dynamics group. However, in this case this parameter had little impact on the Pearson correlation coefficient at the time points checked. This is reasonable since the

$[Fe(Iqsal)_2]^+$ cations and $[Ni(dmit)_2]^-$ anions are much larger so contribute much more to the electron scattering. The contribution from $CH_3CN{\cdot}H_2O$ is negligible.

In conclusion, we found that a simple model with just two parameters, a change in the $[Fe(Iqsal)_2]^+$ cation and a change in the $[Ni(dmit)_2]^-$ anions provided the best explanation of the UED data as presented in the main article.

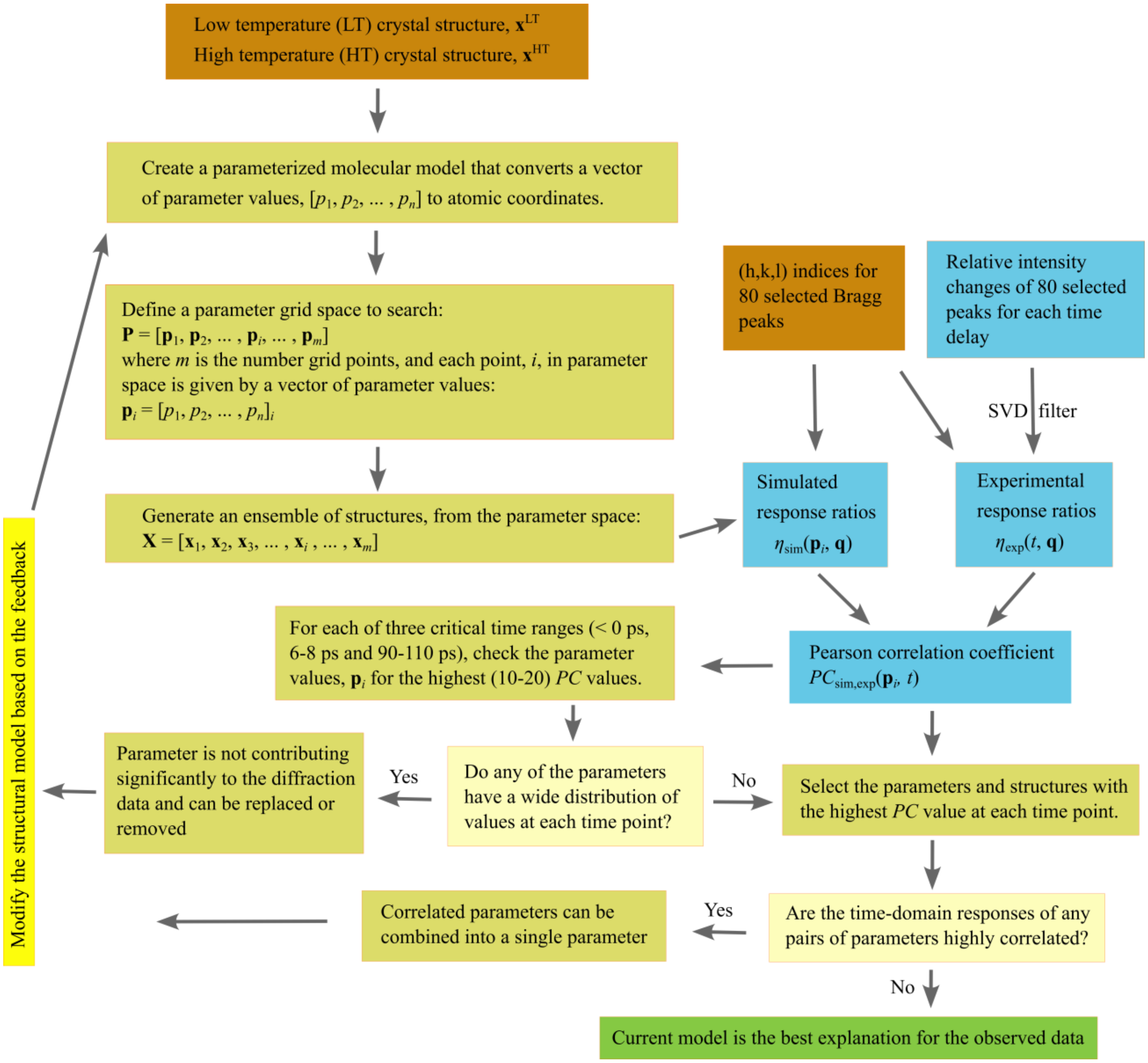

Supplementary Fig. 10 | Workflow for fitting the parameterized molecular model to the UED data. Details of the parameterization are provided in Supplementary Method 6.

Supplementary Method 7 | The interaction energy calculation between the $[Ni(dmit)_2]^-$ dimer

The interaction energies between the $[Ni(dmit)_2]^-$ dimer were computed by single point calculations at the B3LYP functional[11,12] using the implementation of the D3 version of Grimme's dispersion with Becke-Johnson damping[13] and counterpoise corrections[14,15] for basis set superposition error, using the Gaussian 16 program package[16]. The Wachters-Hay basis set for Ni atom,[17,18] and the 6-311+G(d,p) basis set for C[19,20] and S atoms[21–23] were used. The atomic coordinates of the $[Ni(dmit)_2]^-$ anion were taken from the refined structural data from UED at each time delay.

Supplementary Discussion 1 | Photoinduced dynamics of Fe(III) spin crossover

Photoinduced dynamics of $Fe^{(III)}$ SCO complexes have been studied by time-resolved optical spectroscopy[24–26] and time-resolved X-ray diffraction[26,27]. After photoexcitation of the LMCT transition, the molecule is excited from an initial low-spin (LS) state to the LMCT manifold; it relaxes through intersystem crossing and arrives on a HS state within 200 fs; this vibrationally hot HS molecule undergoes rapid intramolecular vibrational energy redistribution (IVR) to a fully relaxed HS state[24,25]. After the initial SCO dynamics, it has been reported that a second increase of the HS fraction occurs in nanocrystals with high fluence. It is assigned as elastic step proceeding with the establishment of mechanical equilibrium with the environment[25–27].

Supplementary Discussion 2 | Assignment of spectral features of mid-infrared vibrational spectroscopy at ~1350 and 1450 cm^{-1}.

To assign the MIR spectral features of **1** at ~1350 and 1450 cm^{-1}, we performed a series of static MIR spectral measurements and quantum chemistry calculations of **1** and its reference samples.

For the MIR feature, as shown in Supplementary Fig. 11, we measured the OD spectra in MIR range of the film of **1** and KBr pellets of two reference samples ($[Fe(Iqsal)_2](NO_3)$, reference of monovalent $[Fe(Iqsal)_2]^+$ cation, and $(TBA)[Ni(dmit)_2]$, for monovalent $[Ni(dmit)_2]^-$ anion) at room temperature. By comparing the spectra of **1** to the spectra of two reference samples, it is clear that the peak at ~1350 cm^{-1} is contributed by the $[Ni(dmit)_2]^-$ anions. This peak has been frequently identified as the C=C stretching mode on the dmit ligands[28–31]. On the other hand, the MIR feature at ~1450 cm^{-1} is mainly contributed by the $[Fe(Iqsal)_2]^+$ cations.

To further identity the mode related these features, we measured static MIR reflectivity measurements of the bulk crystal of 1 at 30 K (LT phase) and 160 K (HT phase), and performed density functional theory (DFT) calculations of the vibrational modes of the $[Fe(Iqsal)_2]^+$ cations at LS and HS in Supplementary Fig. 12. The vibrational modes of the LS and HS states of $[Fe(Iqsal)_2]^+$ cations were computed using the Gaussian 16 program package, starting from geometry-optimized molecules with B3LYP functional using the Wachters-Hay basis set for the Fe atom, the Stuttgart PLC ECP (SDD) basis set for the I atom, and the 6-31G(d,p) basis set for the H, C, N, and O atoms. The calculated vibrational spectra in the LS and HS states match with the experimental measurement. The IR feature at ~1450 cm^{-1} is indeed a coupled stretching mode of C-C bonds in salicylaldimine rings of the $[Fe(Iqsal)_2]^+$ ligands (Supplementary Mov. 1) and its intensity changes due to the SCO phenomenon.

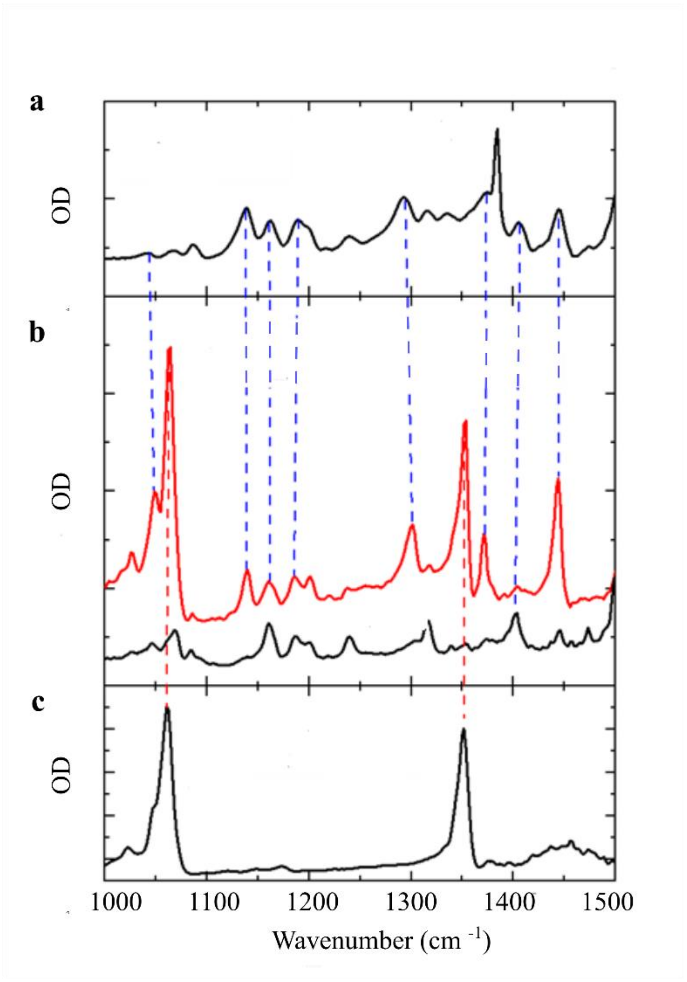

Supplementary Fig. 11 | Static MIR absorption measurements of the 1 film and KBr pellets of two reference samples ([Fe(Iqsal)$_2$](NO$_3$) and (TBA)[Ni(dmit)$_2$]) at room temperature. a, Static MIR absorption spectra of the [Fe(Iqsal)$_2$](NO$_3$) KBr pellet at room temperature. **b,** Static MIR absorption spectra of the film of **1** with $E||b$ (red) and $E||c$ (black) polarization at room temperature. **c,** Static MIR absorption spectra of (TBA)[Ni(dmit)$_2$] at room temperature. Dashed red and blue lines are merely the guide to the eyes. By comparing the spectra of **1** to the spectra of two reference samples, it is clear that the vibrational peak at ~1350 cm^{-1} is contributed by the [Ni(dmit)$_2$]$^-$ anions, and the MIR feature at ~1450 cm^{-1} is contributed by the [Fe(Iqsal)$_2$]$^+$ cations.

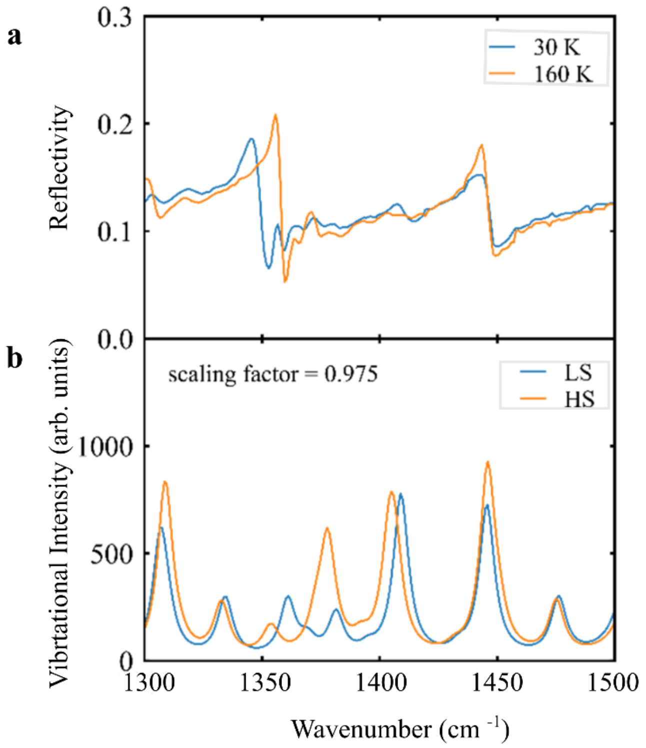

Supplementary Fig. 12 | Static MIR measurements of the 1 bulk crystal in LT and HT phases and DFT calculation of vibrational modes of the $[Fe(Iqsal)_2]^+$ cations in LS and HS states. a, Static MIR measurements of the **1** film in LT (30 K) and HT (160 K) phases. **b,** DFT calculation of modes of the $[Fe(Iqsal)_2]^+$ cations in LS and HS states. The normalized factor is 0.975.

Supplementary Discussion 3 | Impact of volume expansion in dynamics

Here we discuss the volume expansion associated with acoustic phonon and its impact on the dynamics observed in this work. It has been repeatedly reported that after the initial SCO dynamics, the Fe-ligand expansion at the molecular scale produces a strained HS state structure and the unit cell volume is constrained in the first 10 ps[25,27]. In tens of ps timescale, most of absorbed energy is dissipated as energy transfer from photoexcited molecules to the lattice[27], since the absorbed photon energy is much higher than the energy difference between HS and LS and energy difference between SD and WD (tens of meV). The volume starts to expand through a coherent propagative process as elastically driven expansion (strain waves)[27]. This process is called global volume expansion. In the UED and TA experiments of this work, this global volume expansion is expected to continue with a time scale of 40 ps depending on the sample thickness (~ 100 nm) in these two experiments. This time scale corresponds to a 1/4 period of acoustic breathing of the sample, and we observed oscillating features with approximately 160 ps period in the TA data (Fig. 2d). Therefore, there is a possibility that the observed dimer softening process with time constant of ~50 ps might be a consequence of global volume expansion. However, we also observed a similar 50 ps relaxation dynamics in the MIR experiment that was performed with a 15 μm thick sample,

indicating that the observed dynamics are independent of thickness and appear to be a result of local phenomena rather than global volume expansion.

Another discussion is whether dimer softening can be induced by the global volume expansion. The extent of volume expansion and strain waves is contingent on the excitation strength. Previous studies that have reported the global volume expansion driving phase transitions as an elastic step have been performed at much higher fluences of 38 mJ/cm^2 in the SCO crystal[25,27] and 100 mJ/cm^2 in the metal oxide crystal[32] on the similar time scale of the dimer softening we observed. In contrast, our study employed lower fluences ranging from 0.55 to 1.7 mJ/cm^2. We think the phase transition as an elastic step cannot occur with our experimental condition.

Supplementary Discussion 4 | Influence of volume expansion in UED data

Lattice expansion could potentially be important in UED data, but we believe it is too small to be observed here. First of all, we did not see any shift of the Bragg peaks in the UED measurement which would be a clear indication of a change in volume.

To estimate the maximum temperature increase ($\Delta T_{lattice}$) we adopt a reported method[27] to yield a value of approximately 70 K, resulting from the laser power in our UED measurements. Note that this estimate relies on the deposited laser power, the heat capacity of a comparable sample, and assumes full energy conversion to heat. It does not consider radiative losses, and it is likely an upper limit.

Next, we examined whether the lattice parameter change due to a 70 K temperature increase is detectable in our UED setup. In our UED experiment, the average peak width in reciprocal space is approximately 0.21 Å^{-1} (equivalent to 7 pixels of the CCD camera). Based on temperature dependence crystallographic data[1], we calculated the changes of volume size and unit cell parameters by interpolating two low temperature lattice constants. The expansion along each axis is: b-axis (0.46 Å, 1.13 %); a-axis (0.014 Å, 0.14 %) and c-axis (−0.007 Å, −0.07 %). The shift along the b axis would therefore be expected to be the largest, but would still be very small, for example, the Bragg peak (0 10 0) is expected to exhibit a 0.014 Å^{-1} shift, which is approximately 6 % of the peak width and less than one pixel of the detector. Such changes might be observable with higher signal-to-noise ratios, for higher resolution data in terms of reciprocal space, or samples with larger thermal (or elastic) volume expansion effects but are not expected to be observable in this case.

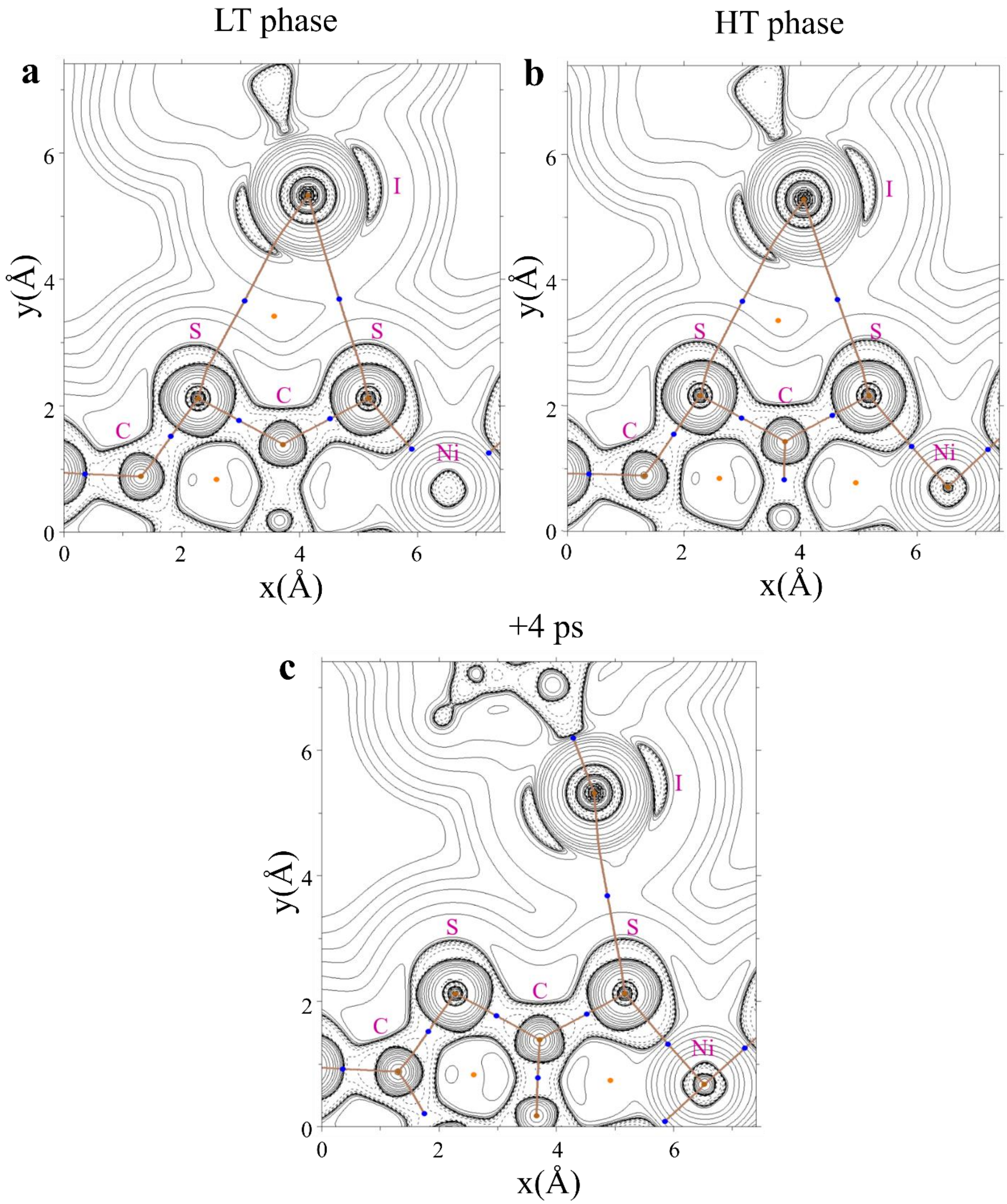

Supplementary Fig. 13 | Bond Critical Points in Laplacian of electron density in the plane of the halogen bond. **a**, Laplacian of electron density in the LT phase (105 K). **b**, Laplacian of electron density in the HT phase (175 K). **c**, Laplacian of electron density of a selected time-delay (4 ps). Blue points represent the bond critical points, which refer to saddle points between the maxima. Brown lines show the bonds. The orange points represent the ring critical points, which

refer to a point of the minimum electron density within the ring surface and a maximum on the ring line.

Supplementary References

1. Fukuroi, K. *et al.* Synergistic spin transition between spin crossover and spin-peierls-like singlet formation in the halogen-bonded molecular hybrid system: $[Fe(Iqsal)_2][Ni(dmit)_2]\cdot CH_3CN\cdot H_2O$. *Angew. Chemie - Int. Ed.* **53**, 1983–1986 (2014).

2. Gawelda, W. *et al.* Ultrafast nonadiabatic dynamics of [FeII(bpy)3] 2+ in solution. *J. Am. Chem. Soc.* **129**, 8199–8206 (2007).

3. Van Stokkum, I. H. M., Larsen, D. S. & Van Grondelle, R. Global and target analysis of time-resolved spectra. *Biochim. Biophys. Acta - Bioenerg.* **1657**, 82–104 (2004).

4. Liu, L. C. *Chemistry in Action : Making Molecular Movies with Ultrafast Electron Diffraction and Data Science*. (Springer Cham, 2020).

5. Field, R., Liu, L. C., Gawelda, W., Lu, C. & Miller, R. J. D. Spectral Signatures of Ultrafast Spin Crossover in Single Crystal $[Fe^{II}(bpy)_3](PF_6)_2$. *Chem. - A Eur. J.* **22**, 5118–5122 (2016).

6. Schmøkel, M. S., Kamiski, R., Benedict, J. B. & Coppens, P. Data scaling and temperature calibration in time-resolved photocrystallographic experiments. *Acta Crystallogr A* **66**, 632–636 (2010).

7. Carbone, F., Yang, D.-S., Giannini, E. & Zewail, A. H. Direct role of structural dynamics in electron-lattice coupling of superconducting cuprates. *Proc. Natl. Acad. Sci. U. S. A.* **105**, 20161–20166 (2008).

8. Jiang, Y. *et al.* Structural Dynamics upon Photoexcitation in a Spin Crossover Crystal Probed with Femtosecond Electron Diffraction. *Angew. Chemie - Int. Ed.* **56**, 7130–7134 (2017).

9. Gao, M. *et al.* Mapping molecular motions leading to charge delocalization with ultrabright electrons. *Nature* **496**, 343–6 (2013).

10. Jiang, Y. *et al.* Direct observation of nuclear reorganization driven by ultrafast spin transitions. *Nat. Commun.* **11**, 1530 (2020).

11. Becke, A. D. A new mixing of Hartree–Fock and local density-functional theories. *J. Chem. Phys.* **98**, 1372–1377 (1993).

12. Lee, C., Yang, W. & Parr, R. G. Development of the Colle-Salvetti correlation-energy formula into a functional of the electron density. *Phys. Rev. B* **37**, 785–789 (1988).

13. Grimme, S., Ehrlich, S. & Goerigk, L. Effect of the damping function in dispersion corrected density functional theory. *J. Comput. Chem.* **32**, 1456–1465 (2011).

14. Boys, S. F. & Bernardi, F. The calculation of small molecular interactions by the differences of separate total energies. Some procedures with reduced errors. *Mol. Phys.*

19, 553–566 (2006).

15. Simon, S., Duran, M. & Dannenberg, J. J. How does basis set superposition error change the potential surfaces for hydrogen-bonded dimers? *J. Chem. Phys.* **105**, 11024–11031 (1996).

16. Frisch, M. J.; Trucks, G. W.; Schlegel, H. B.; Scuseria, G. E.; Robb, M. A.; Cheeseman, J. R.; Scalmani, G.; Barone, V.; Petersson, G. A.; Nakatsuji, H.; Li, X.; Caricato, M.; Marenich, A. V.; Bloino, J.; Janesko, B. G.; Gomperts, R.; Mennucci, B.; Hratch, D. J. Gaussian 16, Revision C.01. (2016).

17. Wachtees, A. J. H. Gaussian Basis Set for Molecular Wavefunctions Containing Third-Row Atoms. *J. Chem. Phys.* **52**, 1033–1036 (1970).

18. Hay, P. J. Gaussian basis sets for molecular calculations. The representation of 3d orbitals in transition-metal atoms. *J. Chem. Phys.* **66**, 4377–4384 (1977).

19. Krishnan, R., Binkley, J. S., Seeger, R. & Pople, J. A. Self-consistent molecular orbital methods. XX. A basis set for correlated wave functions. *J. Chem. Phys.* **72**, 650–654 (1980).

20. Clark, T., Chandrasekhar, J., Spitznagel, G. W. & Schleyer, P. V. R. Efficient diffuse function-augmented basis sets for anion calculations. III. The 3-21+G basis set for first-row elements, Li–F. *J. Comput. Chem.* **4**, 294–301 (1983).

21. McLean, A. D. & Chandler, G. S. Contracted Gaussian basis sets for molecular calculations. I. Second row atoms, Z=11–18. *J. Chem. Phys.* **72**, 5639–5648 (1980).

22. Francl, M. M. *et al.* Self-consistent molecular orbital methods. XXIII. A polarization-type basis set for second-row elements. *J. Chem. Phys.* **77**, 3654–3665 (1982).

23. Spitznagel, G. W., Clark, T., von Ragué Schleyer, P. & Hehre, W. J. An evaluation of the performance of diffuse function-augmented basis sets for second row elements, Na-Cl. *J. Comput. Chem.* **8**, 1109–1116 (1987).

24. Bertoni, R. *et al.* Femtosecond spin-state photo-switching dynamics in an Fe^{III} spin crossover solid accompanied by coherent structural vibrations. *J. Mater. Chem. C* **3**, 7792–7801 (2015).

25. Bertoni, R. *et al.* Elastically driven cooperative response of a molecular material impacted by a laser pulse. *Nat. Mater.* **15**, 606–610 (2016).

26. Lorenc, M. *et al.* Successive dynamical steps of photoinduced switching of a molecular Fe(III) spin-crossover material by time-resolved x-ray diffraction. *Phys. Rev. Lett.* **103**, 028301 (2009).

27. Volte, A. *et al.* Dynamical limits for the molecular switching in a photoexcited material revealed by X-ray diffraction. *Commun. Phys.* **5**, 168 (2022).

28. Ramakumar, R., Tanaka, Y. & Yamaji, K. Electron-phonon interactions, pair-transfer processes, and superconductivity in $TTF[Ni(dmit)_2]_2$. *Phys. Rev. B* **56**, 795 (1997).

29. Pokhodnya, K. I. *et al.* Infrared and Raman properties of $[M(dmit)_2]$ (M=Ni, Pd) based

compounds. *Synth. Met.* **103**, 2016–2019 (1999).

30. Liu, H., Tanner, D., Pullen, A., Abboud, K. & Reynolds, J. Optical and transport studies of Ni(dmit)$_2$-based organic conductors. *Phys. Rev. B* **53**, 10557 (1996).

31. Tamura, M. *et al.* Spectroscopic evidence for the low-temperature charge-separated state of [Pd(dmit)$_2$] salts. *Chem. Phys. Lett.* **411**, 133–137 (2005).

32. Mariette, C. *et al.* Strain wave pathway to semiconductor-to-metal transition revealed by time-resolved X-ray powder diffraction. *Nat. Commun.* **12**, 1239 (2021).
