# Supplementary material for: Direct observation of photoinduced sequential spin transition in a halogen-bonded hybrid system by complementary ultrafast optical and electron probes
Source: Nat Commun. 2024 Jun 4;15:4604. doi: 10.1038/s41467-024-48529-1 (PMC11150260; doi:10.1038/s41467-024-48529-1)
Supplement: Supplementary file 3 — Description of Additional Supplementary Files [file 41467_2024_48529_MOESM3_ESM.pdf]

Description of Additional Supplementary Files

Supplementary Data 1: **Optimized atomic coordinates of low-spin state structure.**

Supplementary Data 2: **Optimized atomic coordinates of high-spin state structure.**

Supplementary Movie 1: **Vibrational modes of the $[Fe(Iqsal)_2]^+$ cations at LS at ~1450 cm^{-1}.** The IR feature at ~1450 cm^{-1} is indeed a coupled stretching mode of C-C bonds in salicylaldimine rings of the $[Fe(Iqsal)_2]^+$ ligands

Supplementary Movie 2: **Vibrational modes of the $[Fe(Iqsal)_2]^+$ cations at HS at ~1450 cm^{-1}.** The IR feature at ~1450 cm^{-1} is indeed a coupled stretching mode of C-C bonds in salicylaldimine rings of the $[Fe(Iqsal)_2]^+$ ligands

Supplementary Movie 3: **Time resolved Laplacian of electron density in the plane of I2-S6-S8.** I2 is Iodine atom on $[Fe(Iqsal)_2]^+$ cation. S6 and S8 are sulfur atoms on the $[Ni(dmit)_2]^-$ anion.

Supplementary Movie 4: **Molecular movie of the photoinduced dynamics.** Two structural dynamics groups are presented, and the movie shows the observed motions over 100 ps in steps of 500 fs.
