# Supplementary figures and images for: Direct observation of photoinduced sequential spin transition in a halogen-bonded hybrid system by complementary ultrafast optical and electron probes
Source: Nat Commun. 2024 Jun 4;15:4604. doi: 10.1038/s41467-024-48529-1 (PMC11150260; doi:10.1038/s41467-024-48529-1)

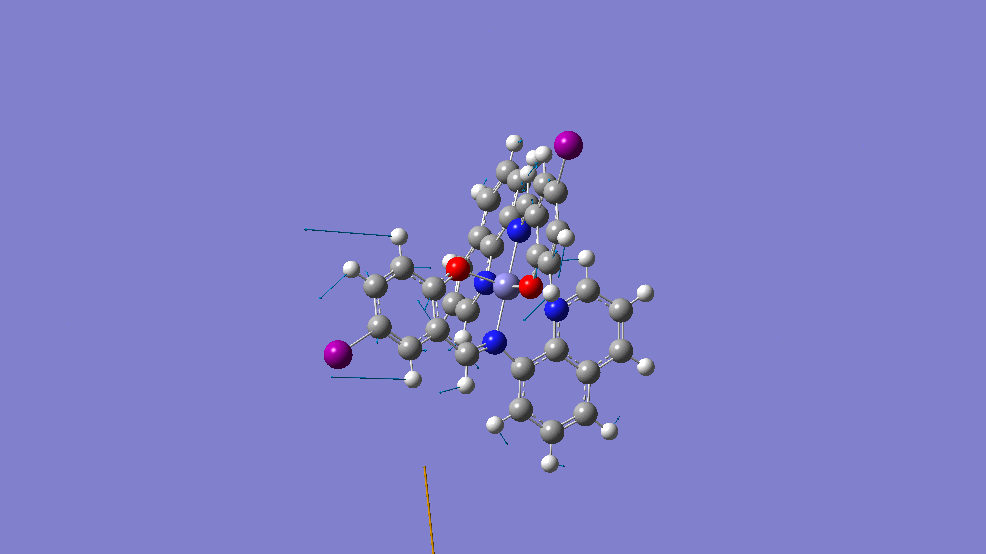

Supplement: Supplementary file 6 — Supplementary Movie 1 [file 41467_2024_48529_MOESM6_ESM.gif]

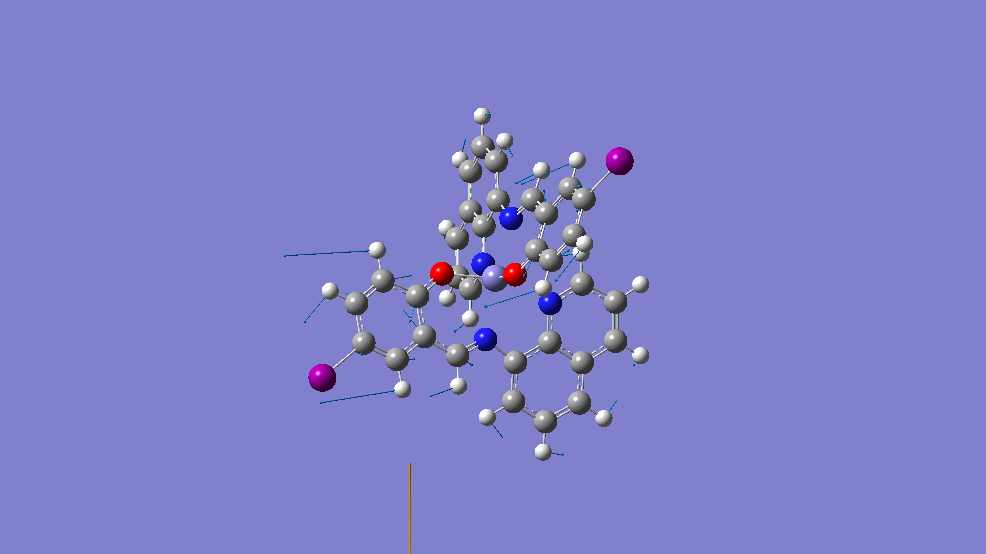

Supplement: Supplementary file 7 — Supplementary Movie 2 [file 41467_2024_48529_MOESM7_ESM.gif]

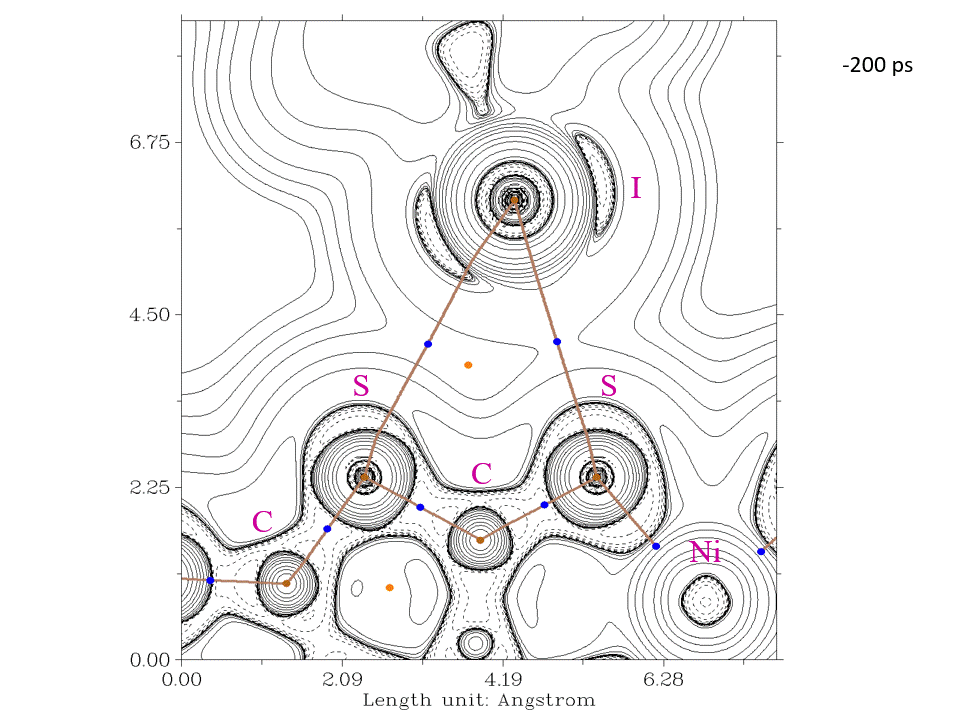

Supplement: Supplementary file 8 — Supplementary Movie 3 [file 41467_2024_48529_MOESM8_ESM.gif]
